# One-Step Solid Extraction for Simultaneous Determination of Eleven Commonly Used Anticancer Drugs and One Active Metabolite in Human Plasma by HPLC-MS/MS

**DOI:** 10.1155/2018/7967694

**Published:** 2018-06-24

**Authors:** Shouhong Gao, Zhengbo Tao, Jingya Zhou, Zhipeng Wang, Yunlei Yun, Mingming Li, Feng Zhang, Wansheng Chen, Yejun Miao

**Affiliations:** ^1^Department of Pharmacy, Changzheng Hospital, Second Military Medical University, Shanghai 200003, China; ^2^Department of Orthopaedics, First Affiliated Hospital, China Medical University, 155 Nan Jing Bei Street, Shenyang, Liaoning 110001, China; ^3^Department of Psychiatry, Ankang Hospital, Ningbo, Zhejiang 315000, China

## Abstract

Therapeutic drug monitoring for anticancer drugs could timely reflect *in vivo* drug exposure, and it was a powerful tool for adjusting and maintaining drug concentration into a reasonable range, so that an enhanced efficacy and declined adverse reactions could be achieved. A liquid chromatography-tandem mass spectrometry method had been developed and fully validated for simultaneous determination of paclitaxel, docetaxel, vinblastine, vinorelbine, pemetrexed, carboplatin, etoposide, cyclophosphamide, ifosfamide, gemcitabine, irinotecan, and SN-38 (an active metabolite of irinotecan) in human plasma from cancer patients after intravenous drip of chemotherapy drugs. One-step solid-phase extraction was successfully applied using an Ostro sample preparation 96-well plate for plasma samples pretreated with acetonitrile containing 0.1% formic acid. Chromatographic separation was achieved on an Atlantis T_3_-C_18_ column (2.1 × 100 mm, 3.0 *μ*m) with gradient elution using a mobile phase consisting of acetonitrile and 10 mM ammonium acetate plus 0.1% formic acid in water, and the flow rate was 0.25 mL/min. The Agilent G6410A triple quadrupole liquid chromatography-mass spectrometry system was operated under the multiple reaction monitoring mode with an electrospray ionization in the positive mode. Linear range was 25.0–2500.0 ng for paclitaxel, 10.0–1000.0 ng for docetaxel and SN-38, 100.0–10000.0 ng for vinorelbine and pemetrexed, 10.0–10000.0 ng for vinblastine and irinotecan, 1.0–1000.0 ng for cyclophosphamide and ifosfamide, 50.0–5000.0 ng for carboplatin, etoposide, and gemcitabine. Linearity coefficients of correlation were >0.99 for all analytes. The intraday and interday accuracy and precision of the method were within ±15.0% and less than 15%. The mean recovery and matrix effect as well as stability of all the analytes ranged from 56.2% to 98.9% and 85.2% to 101.3% as well as within ±15.0%. This robust and efficient method was successfully applied to implement therapeutic drug monitoring for cancer patients in clinical application.

## 1. Introduction

Cancer was gradually becoming an heavy burden for both developed and developing countries, and its incidence and mortality had increased awfully in recent years along with the aging of people and the deterioration of environment as well as the changing of lifestyles, for example, smoking, alcohol abuse, and massive intake of red meat. There were about 14.1 million new cancer cases and 8.2 million deaths in the worldwide in 2012 according to the GLOBOCAN report [1]. A large amount of costs must be paid on the prevention and treatment of cancer in the developed countries, and an upward trend was emerging in the developing countries.

In clinical application, the ways for treatment of cancer often included radiotherapy, chemotherapy, and surgery. Among that, chemotherapy was a potent and adjuvant method served for the surgery and sporadic cancer cells eradication. In recent years, enormous progress had been made in chemotherapy agents, and drug combinations containing multiple cytotoxic anticancer agents or biotherapy occupied the dominant status and yielded a good paradigm for cancer treatment [2, 3]. Drug combinations generally aimed at several targets or pathways to deal with the heterogeneity or multiprocess of cancer, for instance, invasion, growth, and metastasis [4]. Enhanced efficacy and shortened therapy period usually could be obtained by prescribing drug combinations, but the adverse reactions were extended based on each agent in the drug combination. Also, the therapeutic efficacy and adverse reactions were often concentration dependent [5].

The area under the concentration-time curve, steady-state concentration, and concentration over the threshold had a close relationship with the therapeutic results, and they had been considered as potent biomarkers for the individualized treatment in recent years [6]. Concentrations which are in the therapeutic window could obviously increase the responses and sometimes decrease the adverse reactions [7–10]. To guarantee the steady state of drug concentration and its location in the therapeutic window, it was necessary to carry out therapeutic drug monitoring (TDM) for the drug combinations so that clinicians could adjust the therapeutic regimen precisely [11]. TDM could be defined as the measurement of drugs in biological samples to individualize treatment by adjusting the therapeutic regimen. Many clinical practices have proved that TDM was an effective method to improve efficacy and/or reduce the toxicity [12, 13]. Many studies on LC-MS/MS method for quantification of cyclophosphamide, ifosfamide, irinotecan, etoposide, gemcitabine, carboplatin, pemetrexed, paclitaxel, vinblastine, and vinorelbine in human blood had been reported in the latest five years [14–24]. Some of them had shown advantages of shorter run time, simpler sample pretreatment, much lower limit of quantification, or more extensive linear range. But only few methods were developed for simultaneous quantification of drug combinations.

A HPLC-MS/MS method for simultaneous determination of seven anticancer agents had been developed and reported in our laboratory, but two steps of extraction hindered its high throughput in clinical application [25]. Thus, the aim of this study was to (1) optimize the pretreatment procedure for the extraction of anticancer drugs from human plasma and (2) enlarge the scope of clinical application of this method by integrating more anticancer drugs into one method based on our previously reported method. The development and validation of this new method was carried out according to the Chinese Pharmacopeia (vision 2010), and its clinical application was also verified at the same time.

## 2. Materials and Methods

### 2.1. Chemicals and Reagents

**Figure 1:**
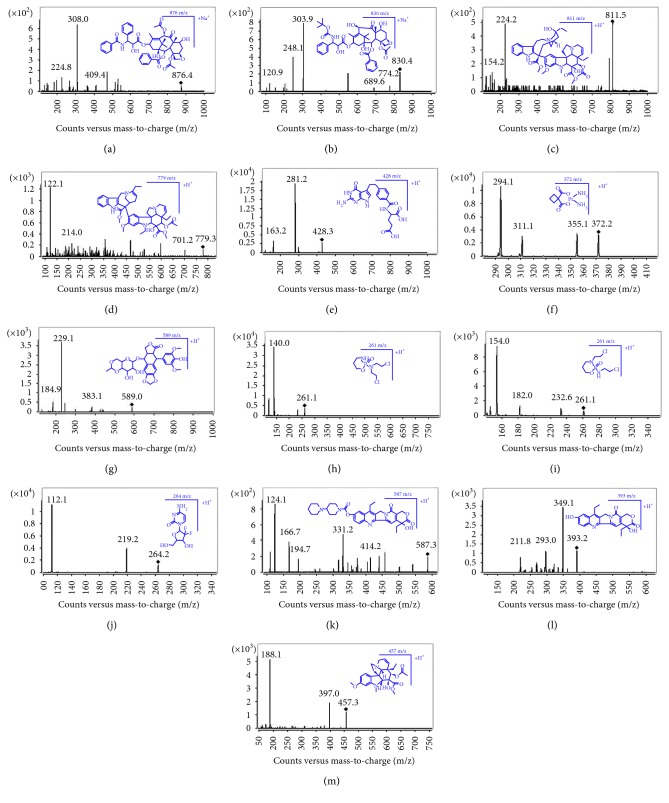
Chemical structures and multiple reaction monitoring ions of paclitaxel (a), docetaxel (b), vinblastine (c), vinorelbine (d), pemetrexed (e), carboplatin (f), etoposide (g), cyclophosphamide (h), ifosfamide (i), gemcitabine (j), irinotecan (k), SN-38 (l), and vindoline ((m), IS).

Paclitaxel, docetaxel, vinblastine, vinorelbine, pemetrexed, carboplatin, etoposide, cyclophosphamide, ifosfamide, gemcitabine, irinotecan, SN-38, and vindoline (internal standard, IS) were purchased from Sigma-Aldrich Corporation (St. Louis, MO, USA). All were corrected for purity and salt forms when weighed or diluted for standard stocks, whose chemical structures are shown in Figure 1. HPLC-grade methanol and acetonitrile were obtained from Merck Company (Darmstadt, Germany). HPLC-grade formic acid, dimethyl sulfoxide (DMSO), and ammonium acetate were purchased from the Tedia Company, Inc. (Fairfield, OH, USA). Ultra-purified water (0.22 *μ*m) was self-made in the laboratory by a Milli-Q reagent water system (Merck KGaA, Darmstadt, Germany) and was used throughout. Waters Ostro 96-well plate (25 mg) was used for sample pretreatment (Waters, Milford, MA, USA). Human blank plasma was donated by Shanghai Red Cross Blood Center (Shanghai, China).

### 2.2. LC-MS/MS Instrumentation

All experiments were carried out on an Agilent 1200 series HPLC system including an online degasser, a quatpump, an autosampler, and a column oven and interfaced to an Agilent 6410A triple-quadrupole mass spectrometer with an electrospray ionization source (Agilent Corporation, Santa Clara, CA, USA). All data were acquired and analyzed using Agilent Masshunter data processing software (version B.01.02, Agilent Corporation, Santa Clara, CA, USA).

### 2.3. Liquid Chromatographic Conditions

The chromatographic separation was achieved on an Atlantis T3-C_18_ analytical column (3.0 *μ*m, 2.1 × 100 mm, Waters, Milford, MA, USA). The column was equilibrated and eluted with a mixed mobile phase consisting of acetonitrile and water containing 0.1% formic acid plus 10 mM ammonium acetate at a flow rate of 0.25 mL/min. The mobile phase was degassed automatically using the online degasser system. Mobile phase A was water containing 0.1% formic acid plus 10 mM ammonium acetate. Mobile phase B was acetonitrile. The gradient variation started with 100% A and maintained for 1 min, then switched to 100% mobile phase B at 1.01 min and lasted until 9 min, and finally switched back to 100% mobile phase A at 9.01 and lasted until 10 min, after which the system was returned to the initial condition. Under these conditions, the analytes coeluted with the IS within 9 min. The column temperature was maintained at 35°C. The injection volume was 10 *μ*L, and the analysis time was 10.0 min.

### 2.4. Mass Spectrometry Conditions

The mass detection was achieved using electrospray ionization in the positive mode with the spray voltage set at 4000 V. Nitrogen was used as nebulizer gas, and nebulizer pressure was set at 40 psi with a source temperature of 105°C. Drying gas (nitrogen) was heated to 350°C and delivered at a flow rate of 10 L/min. High-purity nitrogen was used as collision gas at a pressure of about 0.2 MPa. Quantitation detection was performed in the multiple reaction monitoring (MRM) mode. The peak widths of precursors and product ions were maintained at 0.7 amu at half-height in the MRM mode.

### 2.5. Preparation of Standard and Quality Control (QC) Samples

The stock solutions of paclitaxel, docetaxel, vinblastine, vinorelbine, etoposide, cyclophosphamide, ifosfamide, and vindoline (IS) were individually prepared in methanol, the stock solutions of pemetrexed, carboplatin, and gemcitabine were individually prepared in water, and the stock solutions of irinotecan and SN-38 were prepared in DMSO to obtain final concentrations at 1.0 mg/mL. All stock solutions were stored at −20°C. The stock solution of each analyte was further diluted with 10% methanol (V : V) to obtain a series of work solutions at concentrations of 0.25, 0.5, 1.25, 2.500, 6.25, 12.5, and 25.0 *μ*g/mL for paclitaxel, 0.1, 0.2, 0.5, 1.0, 2.5, 5.0, and 10.0 *μ*g/mL for docetaxel and SN-38, 1.0, 2.0, 5.0, 1.0, 25.0, 50.0, and 10.0 *μ*g/mL for vinorelbine and pemetrexed, 0.1, 0.5, 1.0, 5.0, 10.0, 50.0, and 100.0 *μ*g/mL for vinblastine and irinotecan, 10.0, 50.0, 100.0, 500.0, 1000.0, 5000.0, and 10000.0 ng/mL for cyclophosphamide and ifosfamide, 0.5, 1.0, 2.5, 5.0, 10.0, 25.0, and 50.0 ng/mL for carboplatin, etoposide, and gemcitabine. Calibration standards were prepared by 10 times dilution of the corresponding combined working solutions with blank human plasma to obtain final concentrations in the range of 25.0–2500 ng/mL for paclitaxel, 10.0–1000.0 ng/mL for docetaxel and SN-38, 0.1–10.0 *μ*g/mL for vinorelbine and pemetrexed, 10.0–10000.0 ng/mL for vinblastine and irinotecan, 1.0–1000.0 ng/mL for cyclophosphamide and ifosfamide, and 50.0–5000.0 ng/mL for carboplatin, etoposide, and gemcitabine. Quality control (QC) samples were also prepared in the same way (50.0, 250.0, and 1250 ng/mL for paclitaxel; 20.0, 100.0, and 500.0 ng/mL for docetaxel and SN-38; 200.0, 1000.0, and 5000.0 ng/mL for vinorelbine and pemetrexed; 50.0, 500.0, 5000.0 ng/mL for vinblastine and irinotecan; and 5.0, 50.0, and 500.0 ng/mL for cyclophosphamide and ifosfamide; 100.0, 500.0, and 2500.0 ng/mL for carboplatin, etoposide, and gemcitabine). The QC samples were stored at −20°C and brought to room temperature (25°C) for thawing before being processed.

### 2.6. Sample Pretreatment

Sample pretreatment was performed on Ostro™ 96-well plate to remove phospholipids and proteins (Waters, Milford, MA, USA). An eight-channel 100 *μ*L and 1000 *μ*L pipetting tools (Eppendorf AG, Hamburg, Germany) were utilized for liquid transfer steps. A 100 *μ*L aliquot of samples was added to the designated well followed by adding 20 *μ*L of IS working solution (100 ng/mL). A 300 *μ*L aliquot of acetonitrile containing 1% formic acid was added to each well to precipitate the plasma proteins. All the wells were vortex mixed by aspirating 3 times with pipette. The mixtures were pulled through the cartridges to deprive the phospholipids using 96-well positive pressure processor (Waters, Milford, MA, USA) under 60 psi for 5 min. A 1 mL collection plate was used for collecting the eluents. After transferring to Eppendorf tubes, the content was evaporated under a gentle nitrogen stream at 45°C. A 100 *μ*L of the initial mobile phase was added to the tube, and the residual was reconstituted after vortex mixing for 3 min and then centrifuged for 10 min at 2500 ×g. The supernatant was transferred to a 1.5 mL glass autosampler vial with inserts, and 10 *μ*L of the supernatant was injected into the HPLC-MS/MS system for analysis.

### 2.7. Human Sample Collection

The research protocol was reviewed and approved by the Ethical Committee of Changzheng Hospital (Shanghai, China) and carried out in Changzheng Hospital. Informed consent was signed by all the recruited patients. Human venous blood samples were collected in heparinized vacuum tubes and gently placed in an ice bath until centrifugation. Samples for irinotecan (300 mg/m^2^), vinblastine (10 mg/m^2^), vinorelbine (25 mg/m^2^), and cyclophosphamide (1000 mg/m^2^) were obtained at 0 h and 24 h after intravenous infusion. Other samples containing ifosfamide, etoposide, gemcitabine, carboplatin, and pemetrexed were collected at 0 h, 3 h, and 24 h after intravenous infusion at dosages of 1200 mg/m^2^, 80 mg/m^2^, 2200 mg/m^2^, 330 mg/m^2^, and 800 mg/m^2^, respectively. For paclitaxel, venous blood samples were harvested at 0, 6, 24, and 48 h after intravenous infusion at dosages of 210 mg/m^2^, 240 mg/m^2^, or 270 mg/m^2^, while for docetaxel at dosages of 100 or 120 mg/m^2^ samples were collected in the same points as paclitaxel. Plasma samples were immediately separated by centrifugation at 2000 ×g and 4°C for 10 min. Plasma samples were transferred to 1.5 mL Eppendorf tubes and stored at −80°C until analysis. All samples were processed within 1 h.

### 2.8. Method Validation

Method validation including specificity, linearity, precision and accuracy, matrix effect, recovery, and stability was performed according to the Chinese pharmacopeia (version 2010).

For specificity, comparison of responses in spiked and blank samples from at least 6 lots was performed. The responses of interferences not more than 20% for analytes and 5% for IS were acceptable.

Matrix effect and recovery were assessed in three replicates at three concentration levels (low, mid, and high) for paclitaxel (50.0, 250.0, and 1250 ng/mL), docetaxel and SN-38 (20.0, 100.0, and 500.0 ng/mL), vinorelbine and pemetrexed (200.0, 1000.0, and 5000.0 ng/mL), vinblastine and irinotecan (50.0, 500.0, and 5000.0 ng/mL), cyclophosphamide and ifosfamide (5.0, 50.0, and 500.0 ng/mL), and carboplatin, etoposide, and gemcitabine (100.0, 500.0, and 2500.0 ng/mL). The matrix effect was the ratio of peak area in the spiked postextraction samples to the concentration corresponding solvent substituted samples, and the recovery was the ratio of peak area in the spiked samples concentration corresponding spiked postextraction samples. The IS was also assessed in the same way at the concentration of 100 ng/mL.

Interday and intraday precision and accuracy were also assessed in five replicates at three concentration levels (low, mid, and high). Samples were analyzed in three analytical lots in separate days (at least 2 days), and the RSD% for interday and intraday precision not more than 15% was rational. For intraday and interday accuracy, RE% (relative error) within 15% was considered acceptable.

Linearity of each analyte was evaluated in three analytical lots, and calibration curves were regressed from IS-adjusted peak area versus corresponding concentrations in at least six calibration standards using a 1/*χ*^2^ weighted linear least-squares regression model. The LLOQ was set at the lowest point of the calibration curve, which must possess a 10 times response higher than interference, and the deviation for the precision and accuracy should not be more than 20% and should be within ±20%.

Stability including long-term stability (3 months), short-term stability (24 h in an autosampler), and three frozen-thaw cycle stability was evaluated using QC samples at three levels (low, mid, and high). The calibration curve for each analyte was employed to obtain the measuring concentrations, and the deviation from nominal concentration within 15% was conformed to the criterion.

## 3. Results and Discussion

### 3.1. LC-MS/MS Optimization

The chromatographic conditions, especially the composition of the mobile phase and types of columns, were optimized through several tests to achieve good resolution and symmetric peak shapes for analytes and the IS, as well as shorter run time. A number of C_18_ and C_8_ columns, such as Zorbax SB-C_18_, Zorbax SB-C_8_, Xselect/Xbridge-C_18_, C_8_, and Atlantis T3-C_18_, were tested. Elution of pemetrexed, carboplatin, and gemcitabine needed high percentage (at least 80%) of organic solvent in the mobile phase; however, high proportion of organic solvent (85%) caused that the analytes could not be totally separated from the endogenous interfering materials on the SB-C_18_ and C_8_ column. Finally, the Atlantis T3-C_18_ column provided sufficient retention and suitable separation medium for twelve analytes based on its stronger retention ability. Several mobile phases had been tested: 0.05% formic acid, 0.1% formic acid, 0.05% acetic acid, 0.1% acetic acid, 5 mmol/L ammonium acetate, 10 mmol/L ammonium acetate/water solutions in combination with either methanol or acetonitrile. With methanol as organic solvent, moderate peak tailing was observed, while with acetonitrile, split peaks were shown. As the temperature rises up, the resolution improved with a narrower peak width. In conclusion, the most appropriate mobile phase was acetonitrile and 10 mmol/L ammonium acetate plus 0.1% formic acid in water at a flow rate of 0.25 mL/min, and the column temperature was kept at 30°C. The twelve analytes and the IS were at first characterized by full-scan mode and then product ions mode to ascertain their precursor ions and product ions which were utilized for constructing the MRM mode. The full-scan mode showed that the ionization of the analytes was more suitable in the positive mode. The parameters for fragmentor energy and collision energy were optimized and listed in Table 1.

### 3.2. Sample Pretreatment

Due to the complex composition of plasma, a sample pretreatment was often needed to remove protein and other potential interfering materials prior to LC-MS/MS analysis. Liquid-liquid extraction (LLE) with different organic solvents and protein precipitation (PPT) with acetonitrile or methanol were evaluated as sample pretreatment techniques in our earlier studies. Initially, several conventional PPT [26] and LLE procedures were investigated using different extraction solvents (methanol, acetonitrile, or mixed solvent for PPT, ethyl acetate, diethyl ether, *tert*-butyl methyl ether, or mixed solvent for LLE) [27–29], but no satisfactory recovery and strong matrix effect were obtained for all analytes. A previous pretreatment procedure was developed jointly using PPT by methanol for pemetrexed, gemcitabine, carboplatin and LLE by ethyl acetate for irinotecan, cyclophosphamide, ifosfamide, etoposide, and mixed ether-dichloromethane (7 : 3, V : V) for the remaining analytes to maximize recovery and reduce matrix effect of all analytes, but it was an low efficiency way for extraction of analytes. In order to increase sample throughput, the Ostro 96-well phospholipids removal plate was used, and the results displayed a shorter sample preparation time and higher efficiency. Extraction was done by the PPT using acetonitrile as the eluent solvent, and it showed lesser ion suppression compared with methanol.  Table 2 shows that a one-step sample preparation with an Ostro 96-well plate proved to be simple, rapid, and high efficient for all analytes. In addition, phospholipids in matrix were deprived by the sorbent of Ostro, which also decreased the matrix interference.

### 3.3. Method Validation

#### 3.3.1. Specificity

**Figure 2:**
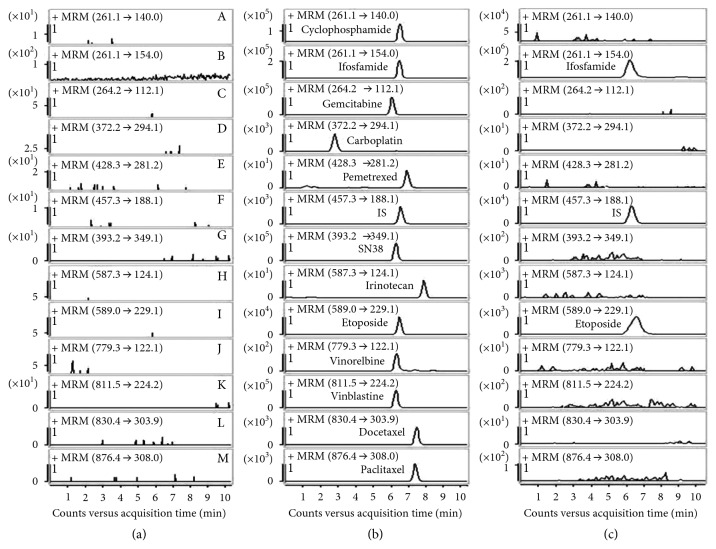
Representative MRM chromatograms of paclitaxel (A), docetaxel (B), vinblastine (C), vinorelbine (D), pemetrexed (E), carboplatin (F), etoposide (G), cyclophosphamide (H), ifosfamide (I), gemcitabine (J), irinotecan (K), SN-38 (L), and vindoline (M, IS). (a) Blank plasma sample, (b) blank plasma sample spiked with twelve analytes at LLOQ and IS, and (c) plasma sample collected from a patient at 6 h after administration of etoposide and ifosfamide at general dose.

Comparison of blank and spiked human plasma chromatograms (Figure 2) indicated no significant interferences at the same retention times of the analytes and the IS.

#### 3.3.2. Matrix Effect and Extraction Recovery

Matrix effect commonly occur due to interfering materials that coexist and coelute in the LC system in the biofluid; generally speaking, the interfering materials that were eluted together with the analytes could influence mostly the response of analytes. The PPT, which usually utilized organic solvent (methanol, acetonitrile, etc.) to strip the proteins in the biofluid by destroying the structure of them, had the lowest ability to eliminate the coexisting interfering materials. Lipids, fats, and other low molecular weight substances could not be striped by the PPT. Part selective extraction of analytes could be completed by an LLE and SPE pretreatment method, but the operation and solvent selection of the two extraction method was relatively complicated. In this study, one-step extraction by an Ostro sample preparation 96-well plate was successfully developed to obtain a high recovery and low matrix effect. The operation simply consisted of PPT and filtration and could be accomplished within 5 min, which promised a high-throughput sample pretreatment. The results showed that a matrix effect ranged from 85.2% to 101.3% and recovery ranged from 56.2% to 98.9%. The matrix effect and recovery were stable and conformed to the pharmacopeia criterion. All data are summarized in Table 3.

#### 3.3.3. Linearity of Calibration Curves and LLOQ

Calibration curves were constructed by plotting the peak area ratios (analyte/IS) of calibration standards versus nominal concentrations from spiked samples. The regression model was constructed based on linear regression with or without intercepts and weighing factors. The best linearity and least-squares residuals for the calibration curves were achieved with a 1/*χ*^2^ weighing factor. The squares of the linear correlation coefficients were all over 0.99. Typical regression equations for the calibration curves were summarized in Table 4. The LLOQ for the determination of paclitaxel, docetaxel, vinblastine, vinorelbine, pemetrexed, carboplatin, etoposide, cyclophosphamide, ifosfamide, gemcitabine, irinotecan, and SN-38 in human plasma, which were defined as the lowest concentration point of the calibration curves, were in accordance with the accuracy within ±20% and precision less than 20%. Five replicates of LLOQ spiked samples for each analyte were analyzed for its precision and accuracy. A signal-to-noise ratio (*S*/*N*) >10 at the LLOQ was observed for all the analytes. These LLOQs were sufficient for clinical drug monitoring of the eleven commonly used anticancer drugs and one active metabolite.

#### 3.3.4. Precision and Accuracy

Three levels of QC samples (low, mid, and high) were chosen to analyze the interday and intraday precision and accuracy. The results showed a good precision and accuracy with intraday and interday precision less than 15% and accuracy within ±15%.  Table 5 summarizes the intraday and interday precision and accuracy for the twelve analytes. All interday and intraday data were acceptable.

#### 3.3.5. Stability

The stability of analytes in long-term cryopreservation, an autosampler, and three frozen-thaw cycles was investigated. The analytes were found to be stable in human plasma stored for 3 months at −20°C and in an autosampler at room temperature for 24 h (<5% reduction). After three freeze-thaw cycles, no obvious reductions (less than 15%) were observed for all the analytes (Table 6).

### 3.4. Application in Determination of Clinical Samples

**Figure 3:**
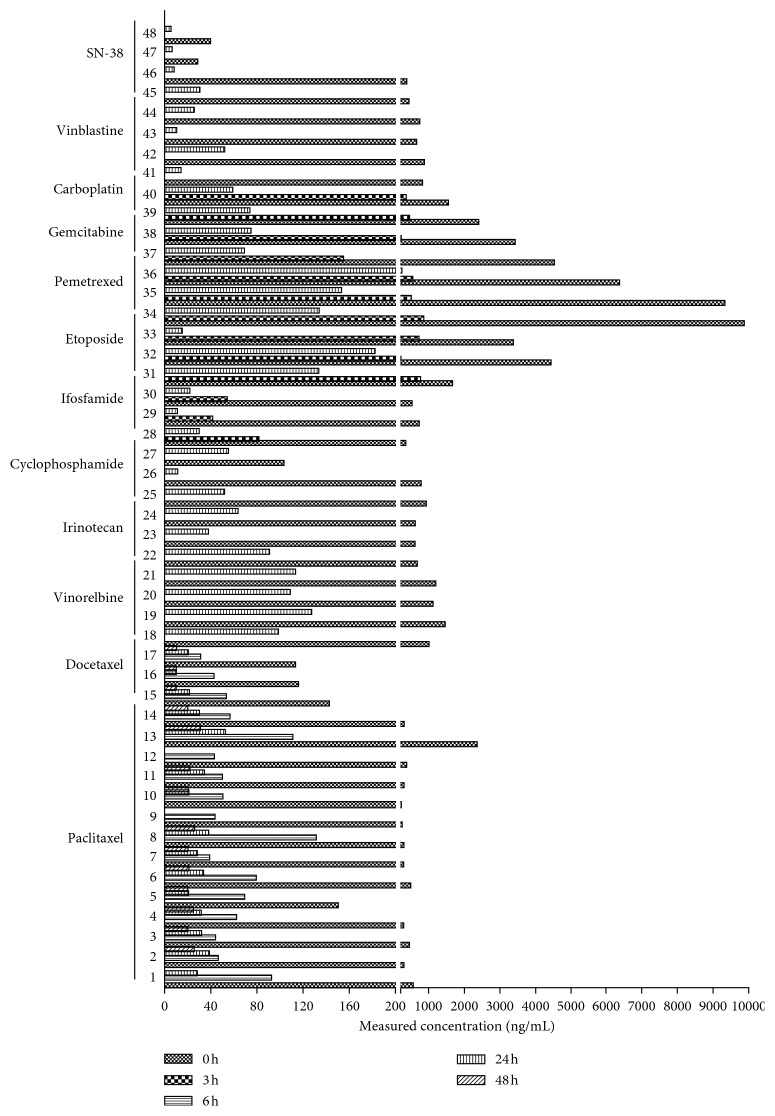
The quantification results of 132 plasma samples from 48 lung cancer patients.

Totally, 132 samples (69 from women and 63 from men) from 48 lung cancer patients were collected. The means (ranges) of age and body weight were 52.5 (18–82) years and 62.0 (51–72) kg, respectively. This entirely validated method was successfully applied for the determination of eleven commonly used anticancer drugs and one active metabolite concentration in the human plasma after administration. The results showed a big difference in patients administered with the same drugs in the same dosage, and the linearity range of this method covered all the concentration variations from different sampling points, which proved an extensive applicability of this method. All data are shown in Figure 3.

## 4. Conclusion

A simple, efficient, and sensitive HPLC-MS/MS method was successfully developed and was suitable for simultaneous determination of eleven commonly used anticancer drugs and one active metabolite in the human plasma from cancer patients. The analytical time was 10 min, and the LLOQ for all analytes was not more than 100 ng/mL. Owing to the application of an Ostro 96-well plate and a special column, this method achieved a simple and efficient sample preparation and separation process, which paved the way for the high throughput and extensive scope in clinical application for routine TDM of anticancer drugs to gain real-time drug exposure so that individualized therapeutic regimen may be promoted.

## Figures and Tables

**Table 1:** Optimized multiple reaction monitoring parameters for the analytes and IS.

Analyte	Precursor ion	Fragmentor energy (V)	Collision energy (eV)	Production ion
Paclitaxel	876.4	250	30	308.0
Docetaxel	830.4	200	20	303.9
Vinblastine	811.5	200	50	224.2
Vinorelbine	779.3	350	40	122.1
Pemetrexed	428.3	80	10	281.2
Carboplatin	372.2	80	10	294.1
Etoposide	589.0	150	10	229.1
Cyclophosphamide	261.1	80	14	140.0
Ifosfamide	261.1	80	20	154.0
Gemcitabine	264.2	80	10	112.1
Irinotecan	587.3	200	40	124.1
SN-38	393.2	150	30	349.1
Vindoline (IS)	457.3	150	20	188.1

**Table 2:** Recovery of all the analytes after extraction with different extraction solvents.

Extraction solvent	Paclitaxel (%)	Docetaxel (%)	Vinblastine (%)	Vinorelbine (%)	Pemetrexed (%)	Carboplatin (%)	Etoposide (%)	Cyclophosphamide (%)	Ifosfamide (%)	Gemcitabine (%)	Irinotecan (%)	SN-38 (%)
Methanol	ND	ND	ND	ND	40.6	50.3	54.4	51.9	45.4	56.3	56.2	65.1
Acetonitrile	24.8	48.8	35.3	44.3	38.2	48.2	57.3	49.5	47.2	57.5	62.8	60.9
Ethyl acetate	53.4	35.3	40.6	57.5	ND	ND	88.0	74.6	71.3	85.3	64.3	68.4
Diethyl ether	93.3	46.9	47.0	82.3	ND	ND	80.4	65.3	63.9	ND	64.0	70.7
*tert*-Butyl methyl ether	76.3	37.0	39.4	68.9	ND	ND	32.2	67.2	67.3	ND	29.3	35.3
Ether-dichloromethane (8 : 2)	80.6	30.9	43.3	88.4	ND	ND	42.7	68.0	66.7	ND	56.7	60.8
Ether-dichloromethane (7 : 3)	52.2	74.5	65.7	80.3	ND	ND	82.4	69.5	60.3	ND	63.1	59.5
Ether-dichloromethane (5 : 5)	69.1	48.4	58.0	67.8	ND	ND	75.9	67.3	59.7	ND	60.7	63.3
Oasis HLB 1 cc (10 mg)	64.0	58.8	56.5	72.3	ND	ND	84.3	91.9	86.4	48.2	82.0	75.8
Ostro 96-well plate	62.4	86.3	90.4	71.2	74.4	73.9	64.7	99.1	77.7	71.4	75.4	68.5

**Table 3:** Matrix effect and recovery of the analytes (*n*=3).

Analyte	Nominal concentration (ng/mL)	Extraction recovery		Matrix effect	
		Mean (%)	RSD (%)	Mean (%)	RSD (%)
Paclitaxel	50.0	60.8	9.5	90.5	6.4
	250.0	66.1	7.2	90.3	3.5
	1250.0	62.3	6.8	92.4	3.7
Docetaxel	20.0	88.2	5.6	91.2	8.8
	100.0	87.3	6.8	89.7	6.4
	500.0	85.5	5.4	92.0	7.1
SN-38	20.0	64.1	7.9	97.3	4.3
	100.0	74.3	6.7	94.3	6.5
	500.0	72.5	5.4	101.9	5.2
Vinorelbine	200.0	69.4	7.9	96.1	1.4
	1000.0	74.6	5.4	101.3	2.1
	5000.0	70.9	7.1	94.9	3.7
Pemetrexed	200.0	70.7	6.0	93.8	6.0
	1000.0	67.7	5.4	99.6	5.8
	5000.0	73.5	5.9	93.4	5.6
Vinblastine	50.0	66.2	11.3	85.2	7.9
	500.0	77.3	9.8	85.7	8.4
	5000.0	80.1	3.8	87.9	4.2
Irinotecan	50.0	60.7	6.6	96.5	9.0
	500.0	64.2	5.9	98.5	8.1
	5000.0	75.1	6.0	97.7	7.4
Cyclophosphamide	5.0	89.9	6.2	82.2	9.9
	50.0	90.1	3.8	90.6	11.3
	500.0	98.9	3.4	90.0	4.4
Ifosfamide	5.0	72.1	5.6	99.1	11.2
	50.0	74.3	2.9	99.5	10.5
	500.0	76.6	4.8	98.0	7.6
Carboplatin	100.0	88.3	8.1	98.0	7.4
	500.0	72.9	4.7	99.7	5.1
	2500.0	72.9	4.9	95.0	7.6
Etoposide	100.0	70.1	6.3	96.9	5.5
	500.0	65.6	8.5	93.7	2.9
	2500.0	64.1	4.9	76.4	4.9
Gemcitabine	100.0	80.8	7.3	99.8	10.6
	500.0	79.3	3.2	99.9	9.8
	2500.0	70.6	8.3	96.3	7.2
IS	100.0	92	5.8	95.7	6.8

**Table 4:** Regression curves and parameters of the analytes (*n*=5).

Analyte	Linearity range (ng/mL)	*y*=*a* *∗* *x*+*b*		*R* ^2^	LLOQ (ng/mL)	LOD (ng/mL)
		*a*	*b*			
Paclitaxel	25.0–2500.0	0.0279	−0.0055	0.9907	25.0	10.0
Docetaxel	10.0–1000.0	0.0109	−0.0008	0.9963	10.0	2.0
SN-38	10.0–1000.0	1.7720	−0.1020	0.9956	10.0	1.0
Vinorelbine	100.0–10000.0	0.2455	−0.2388	0.9939	100.0	50.0
Pemetrexed	100.0–10000.0	0.3559	−0.2553	0.9911	100.0	50.0
Vinblastine	10.0–10000.0	0.6351	−0.0367	0.9944	10.0	2.0
Irinotecan	10.0–10000.0	3.1941	−0.3255	0.9942	10.0	3.0
Cyclophosphamide	1.0–1000.0	3.8840	−0.0072	0.9957	1.0	0.5
Ifosfamide	1.0–1000.0	7.2394	−0.0113	0.9951	1.0	0.5
Carboplatin	50.0–5000.0	0.0388	−0.0046	0.9974	50.0	25.0
Etoposide	50.0–5000.0	0.2673	−0.1549	0.9936	50.0	25.0
Gemcitabine	50.0–5000.0	1.1805	1.1461	0.9919	50.0	25.0

**Table 5:** Intraday and interday precision and accuracy of the analytes in human plasma (*n*=5).

Analyte	Nominal concentration (ng/mL)	Intraday (*n*=5)			Interday (*n*=5)		
		Measured concentration (mean ± SD, ng/mL)	Precision (%RSD)	Accuracy (%RE)	Measured concentration (mean ± SD, ng/mL)	Precision (%RSD)	Accuracy (%RE)
Paclitaxel	50.0	47.78 ± 1.26	2.6	−4.4	47.79 ± 1.69	3.5	−4.4
	250.0	250.06 ± 11.71	4.7	0.0	260.39 ± 11.21	4.3	4.2
	1250.0	1309.21 ± 46.73	3.6	4.7	1278.69 ± 78.67	6.2	2.3
Docetaxel	20.0	18.56 ± 0.40	2.2	−7.2	18.76 ± 0.44	2.3	−6.2
	100.0	96.10 ± 3.69	3.8	−3.9	101.56 ± 5.11	5.0	1.6
	500.0	538.07 ± 21.68	4.0	7.6	518.53 ± 6.09	1.2	3.7
SN-38	20.0	17.56 ± 0.92	5.2	−12.2	16.92 ± 0.42	2.5	−10.4
	100.0	103.26 ± 8.86	8.6	3.3	93.16 ± 1.86	2.0	−6.8
	500.0	546.59 ± 33.33	6.1	9.3	504.15 ± 50.82	10.1	0.8
Vinorelbine	200.0	173.52 ± 1.84	1.1	−13.2	171.59 ± 2.36	1.4	−14.2
	1000.0	1005.74 ± 5.48	0.5	0.6	992.62 ± 4.10	0.4	−0.7
	5000.0	5364.49 ± 2.62	0.1	7.3	5244.73 ± 2.17	0.1	4.9
Pemetrexed	200.0	185.20 ± 13.86	7.5	−7.4	181.32 ± 14.97	8.3	−9.3
	1000.0	951.42 ± 72.67	7.6	−4.9	955.55 ± 39.65	4.1	−4.4
	5000.0	4708.39 ± 445.31	9.5	−5.8	5230.11 ± 168.09	3.2	4.6
Vinblastine	50.0	47.51 ± 0.95	5.4	−5.0	47.08 ± 1.99	11.7	−5.8
	500.0	497.29 ± 9.82	5.0	−0.5	492.92 ± 5.17	2.7	−1.4
	5000.0	5226.19 ± 2.35	0.1	4.5	4990.19 ± 3.67	0.1	−0.2
Irinotecan	50.0	49.89 ± 1.07	5.4	−0.2	47.66 ± 0.94	5.3	−4.7
	500.0	497.83 ± 8.38	4.2	−0.4	495.69 ± 6.01	3.1	−0.9
	5000.0	5379.31 ± 178.38	3.3	7.6	5006.16 ± 196.44	3.9	0.1
Cyclophosphamide	5.0	5.00 ± 0.21	10.4	0.0	4.95 ± 0.23	11.7	−1.0
	50.0	49.27 ± 0.69	3.4	−1.5	48.39 ± 0.31	1.6	−3.2
	500.0	538.97 ± 5.80	1.2	7.8	525.12 ± 9.37	1.9	5.0
Ifosfamide	5.0	4.90 ± 0.10	5.1	−2.0	4.91 ± 0.08	4.0	−1.8
	50.0	49.04 ± 0.51	2.7	−1.9	49.16 ± 0.71	3.7	−1.7
	500.0	526.72 ± 10.80	2.1	5.3	516.36 ± 12.78	2.5	3.3
Carboplatin	100.0	98.57 ± 2.59	2.6	−1.4	103.05 ± 12.69	12.3	3.1
	500.0	479.21 ± 5.69	1.2	−4.2	477.25 ± 11.46	2.4	−4.6
	2500.0	2640.10 ± 40.11	1.5	5.6	2537.97 ± 61.41	2.4	1.5
Etoposide	100.0	99.71 ± 2.56	2.6	−0.3	98.31 ± 2.00	2.0	−1.7
	500.0	482.60 ± 16.72	3.5	−3.5	465.72 ± 14.92	3.2	−6.9
	2500.0	2711.88 ± 59.84	2.2	8.5	2633.31 ± 72.39	2.7	5.3
Gemcitabine	100.0	101.86 ± 7.55	7.4	1.9	86.53 ± 5.64	6.5	−13.5
	500.0	526.06 ± 47.53	9.0	5.2	477.32 ± 33.61	7.0	−4.5
	2500.0	2546.58 ± 100.92	4.0	1.9	2402.05 ± 106.44	4.4	−3.9

^a^RE is calculated as (mean measured concentration/spiked concentration − 1) × 100%.

**Table 6:** Stability of analytes in human plasma (*n*=5).

Analyte	Nominal concentration (ng/mL)	Three freeze-thaw stability		Short-term stability (24 h)		Long-term stability (3 months)	
		Precision (%RSD)	Accuracy (%RE)	Precision (%RSD)	Accuracy (%RE)	Precision (%RSD)	Accuracy (%RE)
Paclitaxel	50.0	1.9	5.9	2.8	6.7	2.4	5.8
	250.0	1.9	3.9	5.6	−1.4	3.6	3.7
	1250.0	4.3	−5.5	4.0	−4.7	3.3	−1.0
Docetaxel	20.0	1.8	6.8	3.1	4.5	2.5	5.3
	100.0	2.1	6.9	4.1	1.6	2.2	3.3
	500.0	1.9	−4.6	2.5	−3.1	2.9	−7.3
SN-38	20.0	**3.6**	−4.7	2.8	2.6	4.6	−3.1
	100.0	**2.9**	4.5	4.6	−3.0	3.5	2.9
	500.0	**4.1**	1.6	3.8	2.7	6.2	3.8
Vinorelbine	200.0	2.0	8.1	3.6	2.8	4.2	6.8
	1000.0	7.2	1.3	4.2	−0.4	6.3	0.9
	5000.0	5.4	3.0	3.3	4.9	3.2	3.5
Pemetrexed	200.0	5.0	2.6	4.7	4.8	4.9	5.2
	1000.0	2.8	2.7	6.5	−1.8	2.4	2.0
	5000.0	3.5	−6.4	4.8	−5.1	4.7	−4.7
Vinblastine	50.0	1.4	0.2	1.9	2.0	4.2	5.1
	500.0	5.0	−2.9	5.5	−3.1	4.0	3.0
	5000.0	3.5	1.5	3.9	2.9	3.2	3.3
Irinotecan	50.0	3.8	5.6	2.7	3.8	3.6	5.6
	500.0	5.5	−3.1	7.4	−1.0	4.5	2.8
	5000.0	2.8	4.3	4.4	1.3	4.0	3.9
Cyclophosphamide	5.0	7.4	1.2	1.9	4.3	5.5	−0.1
	50.0	6.1	3.7	3.0	2.8	3.6	4.8
	500.0	1.8	−4.2	2.4	−4.2	2.1	−3.7
Ifosfamide	5.0	3.5	2.4	2.6	0.2	5.3	3.5
	50.0	4.5	4.2	6.0	3.1	4.6	2.7
	500.0	3.2	−0.2	3.1	0.9	3.5	0.2
Carboplatin	100.0	7.2	4.8	6.9	−1.4	6.2	0.8
	500.0	6.7	2.6	6.6	1.0	7.4	3.1
	2500.0	1.7	−3.0	2.3	−0.7	2.1	−0.9
Etoposide	100.0	4.7	2.7	5.8	0.2	4.7	−0.3
	500.0	3.4	4.4	3.6	−2.5	4.5	3.5
	2500.0	7.3	−1.2	5.9	−4.1	6.5	−2.8
Gemcitabine	100.0	6.7	−1.4	5.6	−2.3	2.5	5.3
	500.0	7.8	−1.3	7.2	−2.0	6.7	−3.2
	2500.0	2.4	−7.2	5.4	−5.1	4.9	−4.0

## Data Availability

The data used to support the findings of this study are available from the corresponding author upon request.
